# Brain areas associated with visual spatial attention display topographic organization during auditory spatial attention

**DOI:** 10.1093/cercor/bhac285

**Published:** 2022-08-16

**Authors:** Tzvetan Popov, Bart Gips, Nathan Weisz, Ole Jensen

**Affiliations:** Methods of Plasticity Research, Department of Psychology, University of Zurich, 1-80502-784644-50205-B15 2TT, Zurich, Switzerland; Department of Psychology, University of Konstanz, Konstanz, Germany; NATO Science and Technology Organization Centre for Maritime Research and Experimentation (CMRE) La Spezia, La Spezia 19126, Italy; Centre for Cognitive Neuroscience and Department of Psychology, University of Salzburg, Salzburg, Austria; School of Psychology, University of Birmingham, Birmingham, UK

**Keywords:** auditory, attention, alpha, spatial orientation, EEG, oscillations

## Abstract

Spatially selective modulation of alpha power (8–14 Hz) is a robust finding in electrophysiological studies of visual attention, and has been recently generalized to auditory spatial attention. This modulation pattern is interpreted as reflecting a top-down mechanism for suppressing distracting input from unattended directions of sound origin. The present study on auditory spatial attention extends this interpretation by demonstrating that alpha power modulation is closely linked to oculomotor action. We designed an auditory paradigm in which participants were required to attend to upcoming sounds from one of 24 loudspeakers arranged in a circular array around the head. Maintaining the location of an auditory cue was associated with a topographically modulated distribution of posterior alpha power resembling the findings known from visual attention. Multivariate analyses allowed the prediction of the sound location in the horizontal plane. Importantly, this prediction was also possible, when derived from signals capturing saccadic activity. A control experiment on auditory spatial attention confirmed that, in absence of any visual/auditory input, lateralization of alpha power is linked to the lateralized direction of gaze. Attending to an auditory target engages oculomotor and visual cortical areas in a topographic manner akin to the retinotopic organization associated with visual attention.

## Introduction

Adaptive behavior in complex environments requires a mechanism enabling the conversion of external events into internal representations in a goal-directed manner. This includes processes to prioritize and direct attention towards goal-relevant stimulus features. In the visual domain, alpha oscillatory activity (8–14 Hz) has been proposed to reflect an “attentional filter” mechanism. When attention is spatially oriented to a particular location in the visual field, alpha power is hemispherically lateralized: it is reduced contralateral to the attended location in a topographic, i.e. retinotopically organized fashion distributed across visual and parietal brain areas (e.g. Kelly et al. 2006; Rihs et al. 2007; Popov et al. 2019).

This “attentional filter” idea has been generalized to the field of auditory spatial attention, adopting the mechanism handling auditory targets (alpha power reduction) and distractors (alpha power increase) (Wostmann et al. 2016; Klatt et al. 2018a, 2018b; Tune et al. 2018; Deng, Choi, et al. 2019; Deng, Reinhart, et al. 2019; Wostmann et al. 2019; Deng et al. 2020; Tune et al. 2021). Spatial analysis and discrimination of auditory input are essential for survival of many living organisms and are central to human spatial orientation and social communication in particular. It is still unclear, to what extent the parietal alpha power modulation is associated with a rather coarse left versus right differentiation, or whether the functional retinotopic organization of visuo-parietal cortex known from studies on visual spatial attention is utilized during auditory spatial attention as well.

The parietal cortex has been established as a region encoding the azimuth of auditory cues (Rauschecker and Tian 2000; Michalka et al. 2016; van der Heijden et al. 2019). In audio-visual spatial cueing paradigms both auditory and parietal areas display lateralization of alpha activity reminiscent of the ones observed in visual attention paradigms (Kerlin et al. 2010; Muller and Weisz 2012; Wostmann et al. 2016; Klatt et al. 2018a, 2018b; Tune et al. 2018; Wostmann et al. 2019; Deng et al. 2020). The notion arose that incoming auditory input might converge on a supramodal representation of space to be integrated with other information and be made accessible to action (Rosenblum et al. 2017; Rauschecker 2018).

In the present report, 24 loudspeakers were horizontally positioned around the participant’s head. An auditory cueing paradigm was used while the participant’s brain activity was monitored by high-density electroencephalography (EEG). The initial research question (H1) was to test the presence or absence of alpha power lateralization akin to the pattern known from visual spatial attention. H2 aims to confirm that these “visual” patterns, induced by auditory spatial cues, can be used to predict the direction of deployed auditory spatial attention. The preregistered hypotheses (https://osf.io/kp95j) were:

There is a spatio-temporal pattern of neural activity in the EEG data that will allow decoding the direction of auditory attention. In support of this hypothesis, we expect that alpha power modulation during the cue-target interval is independent of the sensory domain: the direction of attention cued by auditory stimuli to the left-hand side should prompt modulation of contralateral alpha power over posterior electrodes and vice versa.

Spatial information is encoded following the presentation of auditory cues. In support of this hypothesis, we reasoned that the decoding performance can be compared between periods of spatial auditory cue maintenance and pre cue baseline. Going beyond the left–right stimulus presentation, all additional loudspeaker directions will be considered.

To address the contribution of oculomotor activity, exploratory analyses were conducted utilizing the horizontal electrooculogram (hEOG) during the maintenance interval of an auditory spatial cue. Based on the observations made in this initial experiment, a confirmatory experiment utilizing simultaneous eye tracking and EEG was carried out.

## Materials and methods

### Experiment 1

#### Participants

Thirty-one undergraduates were recruited at the local university (mean age M ± SD 23.6 ± 3.57 years, 18 female). All but one reported no history of neurological and/or psychiatric disorders. All participants gave written informed consent in accordance with the Declaration of Helsinki prior to participation. The study was approved by the University of Konstanz ethics committee.

#### Stimulus material and procedure

**Fig. 1:**
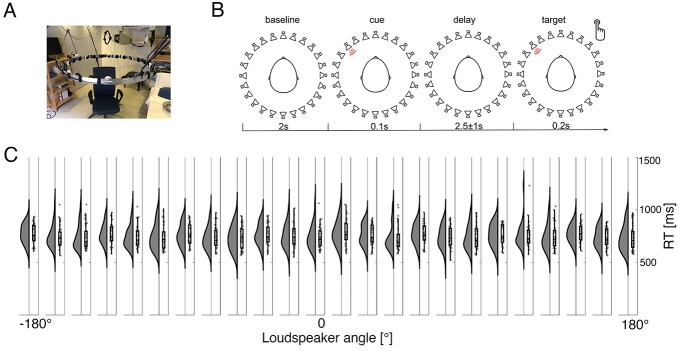
Experimental setup and behavioral results. (A) A photograph of the hardware illustrating an aluminum ring holding the 24 loudspeakers equidistantly placed with an angular distance of 15°. Participants were sitting in a chair with their head positioned in the center of the ring. (B) After a baseline interval of 2 s an auditory cue is presented at one of 24 speaker directions for 100 ms. During the delay interval of 2.5 ± 1 s participant’s maintained the cued direction in memory. After this delay interval, a target syllable “goeb” or “goed” was presented for 200 ms at the cued direction. Participants were asked to indicate via button press, as fast as possible, whether they heard “goeb” or “goed.” (C) Rain cloud plots per loudspeaker direction illustrate a similar distribution of RT across participants.

In an auditory cued spatial attention task, participants were instructed to maintain a comfortable sitting position in the center of an aluminum ring (Fig. 1A). Overall, 24 speaker locations were used within three blocks. After a baseline period (2 s, Fig. 1B), an auditory cue (100 ms duration; 440 Hz) was presented randomly at one of 8 locations (0°, 45°, 90°, 135°, 180°, 225°, 270°, 315°, Fig. 1A). Given an average ear-to-ear distance of ~20 cm, the half wavelength of sound waves below 800 Hz is larger than the head size such that phase delays between both ears can be reliably identified. After a delay interval of 2.5 ± 1 s, during which subjects maintained the cued position, a target syllable (German, “goeb” or “goed”) appeared at that location, embedded in a circular array of 24 speakers mounted at 15° distance on the inner surface of the aluminum ring. Participants indicated via button press whether the target syllable was a “goeb” (index finger, right hand) or a “goed” (middle finger, right hand). All responses were given with the index and middle fingers of the right hand. In each of three blocks 160 trials were presented separated by a short break. Each trial began with the presentation of the “cue,” followed by the delay interval (2.5 ± 1 s), after which the target was presented. Following the button press, the next trial began. The second and third blocks were identical to the first one. The only difference was that the location of cues and targets were shifted with 15° (2nd block) and 30° (3rd block) thereby ensuring a full 24 location circular coverage. Participants were not aware of this change in speaker arrangement. A total of 480 trials (20 per location) were presented. Stimulus presentation was controlled using Presentation software (www.neurobs.com) on a Windows 7 PC.

#### Data acquisition

The EEG was measured in an electrically shielded room using a high-density 256-channel Electrical Geodesics Inc. (EGI) system with a HydroCel Geodesic Sensor Net (GSN; Electrical Geodesics, Inc., Eugene, Oregon, USA). Prior to sampling at 1,000 Hz, the EEG was filtered using a 0.1 Hz high-pass and a 400 Hz low-pass hardware filters. The vertex (Cz) electrode served as a recording reference. All subsequent analyses were performed after converting the data to a common reference and downsampled to 300 Hz. Electrodes around the cheeks and neck were excluded from subsequent analyses. The discarded electorde labels were E253 E241 E242 E243 E244 E245 E248 E246 E249 E252 E247 E250 E254 E67 E68 E61 E54 E46 E37 E32 E31E251 E255 E256 E73 E82 E91 E92 E102 E93 E94 E103 E111 E104 E105 E112 E113 E114 E120 E121 E122 E123 E133 E134 E135 E136 E145 E146 E147 E148 E156 E157 E158 E165 E166 E167 E168 E175 E174 E176 E177 E187 E188 E189 E199 E190 E200 E201 E208 E209 E216 E217 E210 E229 E218 E228 E219 E220 E225 E227 E233 E226 E230 E234 E238 E239 E240 E236 E231 E235 E232 E237 E1 E10 E18 E25. Remaining bad electrodes were identified and removed based on visual expection using the function ft_rejectvisual in FieldTrip. Following EGI acquisition guidelines, electrode impedances were kept below 30kΩ, which is adequate because of the high input impedance of the EGI amplifiers. Standard positions for the present montage were registered to later align with a Montreal Neurological Institute (ICBM 2009a Nonlinear Asymmetric 1 × 1 × 1 mm) template brain (Montreal Neurological Institute, Montreal, Canada http://www.bic.mni.mcgill.ca/ServicesAtlases/ICBM152NLin2009).

#### Neural data analysis

Data analysis was performed using the MATLAB FieldTrip toolbox (Oostenveld et al. 2011). Trials characterized by extreme variance were identified as outliers trough visual inspection and were excluded first. On average 19.4 trials per location (STD = 0.2) were retained for further analyses. After demeaning and removing the linear trend across the session, an independent component analysis (ICA; Jung et al. 2001) was used to remove variance associated with vertical and horizontal eye movements and cardiac activity. Prior to ICA computation, the data were bandpass filtered (1–20 Hz) and the resulting topographies, as well as the unmixing matrix, were used to backproject the data in the original sampling (i.e. 300 Hz).

#### Spectral analysis

Spectral analysis was computed for each trial using the fast Fourier transform (FFT) algorithm based on a sliding window of 500 ms multiplied with a Hanning taper resulting in frequency smoothing of ~3 Hz. Power estimates were calculated for the latency from −1 to 2 s after cue onset in steps of 50 ms and averaged over trials. The estimated frequency range was from 2 to 40 Hz in steps of 2 Hz. Subsequently, power estimates were decomposed into periodic and aperiodic components using the “*specparam*” algorithm (Donoghue et al. 2020). This decomposition allows the identification of oscillatory components in the data such as peaks in the spectrum. Analysis of alpha power lateralization was performed based on the trials with left and right most cueing locations (Fig. 1A, left speakers 6,7,8 and right speakers 18,19,20).

#### Source analysis

Source estimates were computed in the time as well as in the frequency domain. In the frequency domain, an adaptive spatial filtering algorithm was used (dynamic imaging of coherent sources, DICS; Gross et al. 2001). This algorithm uses the cross-spectral density matrix from the EEG data and the lead-field derived from the forward model to construct a spatial filter for a specific location. This matrix was calculated using a multi-taper FFT approach for data in the 0.3–0.8 s interval following the cue onset. Spectral smoothing of ±2 Hz around a 10 Hz center frequency was applied to capture power in the 8–12 Hz (alpha) range. These spectral density matrices and thus the spatial filters were participant-specific and estimated based on all trials and used to estimate the power for the trials with the leftmost (90 ± 15°) and rightmost cues (270 ± 15°). This so-called common spatial filter based on all trials ensures that potential differences in oscillatory power are not due to differences in filter estimates of conditions. A standard forward model was constructed from the MNI ICBM 2009 template brain using the OpenMEEG (Gramfort et al. 2010) implementation of the boundary element method (BEM). A parcellation scheme based on the Desikan-Kiliani atlas was implemented (Desikan et al. 2006). A cortical surface source model was generated consisting of 2,002 dipole locations. The forward solution was applied to all participants and the regularization parameter was set to 5%.

In the time domain, a related spatial filtering algorithm (LCMV, linearly constrained minimum variance) was used(Van Veen et al. 1997). This algorithm uses the covariance matrix of the EEG data to construct a spatial filter for a given location. The covariance matrices for these spatial filters were estimated based on data from all trials within the −0.3 to 1 s interval with respect to cue onset. A 1–20 Hz bandpass filter (one-pass, zero-phase, hamming-windowed sinc finite impulse response (FIR), passband 2–19 Hz, cutoff (−6 dB)) was applied before these operations. Regularization was set to 5%. These filters were applied to the scalp data to derive the time series for a given location. In addition, the parcellation scheme was used to apply the forward encoding model (see next section) on source level data with reasonable amount of “virtual electrodes,” 68 (parcels) rather than 2,002 (dipoles). Namely, single-dipole-specific spatial filters were concatenated across vertices comprising a parcel resulting in 68 multivariate source time series. For each parcel, a principal component analysis was applied to extract the spatially orthogonal and temporally uncorrelated components ordered by the amount of variance explained. The first principal component was selected as the representation of the parcel’s time course of activity.

**Fig. 2:**
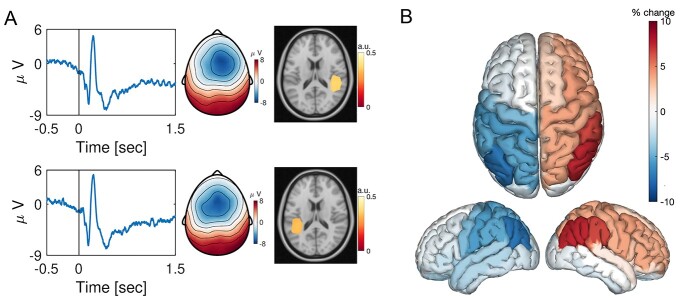
Spatial auditory cues engage the posterior parietal cortex as measured by auditory evoked potentials. (A) Time-series illustrate the auditory potentials averaged across participants at electrode Cz, for the left presented stimuli (top) and right presented stimuli (bottom). Cue onset is denoted at 0 s and baseline correction (−0.5 to 0 s) was applied. The topographies correspond to the latency of the N1 evoked response ~100 ms post cue onset. Cold colors reflect negative and warm colors positive voltage with respect to the pre cue baseline. Source reconstructions of the N1 evoked response confirmed cortical origin within the primary auditory cortex. (B) Source reconstruction of the difference left minus right presented auditory stimuli for the time interval 110–180 ms post cue onset.

Source imaging of N1 evoked activity was carried out following the procedures described in Popov et al. (2018). Due to the location and anatomy of the Heschl’s gyrus as a primary generator of the N1 activity, a cortical surface-based forward model is rather inappropriate. Instead, a forward model using realistically shaped three-layered BEM based on the template magnetic resonance imaging (MRI) described above was calculated. Activity was estimated on a 3D grid of dipole locations with equidistant spacing of 15 mm. Following application of the LCMV algorithm as described above, the absolute value of the dipole moment within the N1 latency (110–180 ms) was averaged. The absolute value was taken due to the arbitrary polarity of the activity reconstructed with beamforming. Source activity was projected onto a structural MRI and thresholded at 80% of maximum for visualization purposes (e.g. Fig. 2A).

#### Forward encoding modeling

Forward encoding modeling followed the procedure described in (Foster et al. 2017) and publicly shared on the https://osf.io/vw4uc/ platform. The analysis was performed on source space data in order to map the activation patterns onto the brain volume. Briefly, the general assumption is that oscillatory power quantified at each electrode reflects the weighted sum of 24 hypothetical responses reflecting the macroscopic manifestation of spatially tuned neuronal populations. Each of these neuronal ensembles is tuned to a different speaker direction (Fig. 1). The EEG data were partitioned into 2 blocks (train and test) with similar trial numbers. A 10-fold random generation of multiple block assignments (e.g. test or train) was utilized and the outcome was averaged over folds. Single-trial alpha power was estimated using a Hilbert transform on the bandpass filtered data (8–12 Hz) identical to the procedures described in (Foster et al. 2016, 2017). Hilbert transformation was used only during forward encoding modeling analyses to stay as close as possible to earlier work (Foster et al. 2016, 2017). All other spectral analyses were done using the sliding-window FFT approach described above. To infer the position of the maintained spatial location from the EEG data, a set of 24 basis functions coding for 24 equally spaced directions between 0° and 360° was constructed first. For each time point, training data *B1* allowed the estimation of weights that approximated the relative contribution of the 24 hypothesized spatial channels (*k*) to the measured scalp data. The response (*R*) of these spatial channels was modeled as a half sinusoid raised to the seventh power, where *R* = sin(0.5*θ*)^7^ with *θ* corresponding to the spatial direction (0° to 359°). Let *B1* (*m* electrodes × *n1* trials) be the signal at each electrode and trial in the training set, *C1* (*k* spatial channels × *n1* trials) the predicted response of each spatial channel, and *W* (*m* electrodes × *k* spatial channels) the weight matrix allowing the linear mapping from “spatial channels space” to electrode space. This mapping was based on a linear model of the form:


(1) \begin{equation*} {C}_1=W{B}_1+\epsilon \end{equation*}

where $\epsilon$ contains (assumed Gaussian) error terms that should be minimized. To this end, ordinary least-squares regression was used to estimate the weight matrix *W (m × k)*:


(2) \begin{equation*} \hat{W}={C}_1{B}_1^T{\left({B}_1{B}_1^T\right)}^{-1} \end{equation*}

Based on this weight matrix and on the test data *B_2_* (*m* electrodes × *n_2_* trials) an estimated response matrix *C_2_* (*k* spatial channels × *n_2_* trials) was calculated:


(3) \begin{equation*} {\hat{C}}_2=\hat{W}{B}_2 \end{equation*}

The estimated responses were circularly shifted such that estimates associated with directions that evoked a response were positioned at 0° of the direction space spanning −180° to 180°. Following this step, an accurate model is characterized by a maximum at 0° and a minimum at −180°/180° (Fig. 4A). In contrast, an inaccurate model fit approximates a flat line. This procedure was performed for each sample point within the −1 to 1 s interval relative to the cue onset. This interval was chosen as such that it encompasses an equal duration of pre- and post-cue latencies of 1 s and avoids ringing artifacts introduced by the band pass filter (i.e. instead of −1.5 to 1.5 s available given a delay interval of 2.5 ± 1 s, −1 to 1 s was chosen). This was repeated until each block had served as a training and test set.

Finally, to interpret the weight matrix *W* in terms of source origin, an activation matrix *A* of a corresponding forward encoding model was computed (Haufe et al. 2014):


(4) \begin{equation*} A={\varSigma}_{B_1}\ {W}^T\ {\varSigma}_{{\hat{C}}_1} \end{equation*}

Here, ${\varSigma}_{B_1}=\operatorname{cov}({B}_1)$ and ${\varSigma}_{\hat{C}_1}=\operatorname{cov}({\hat{C}}_1)=\operatorname{cov}(\hat{W}{B}_1)$ are covariance matrices. The advantage of using *A* instead of the raw weights *W* is that elements of *W* may reflect suppression of “signals of no interest” (Haufe et al. 2014). For example, correlations across sensors in *B*_1_ could be confounded by noise. Therefore, they do not reflect brain activity related to *C*_1_. Transforming to activation patterns *A* mitigates this problem. A graphical illustration of the approach is provided in Supplementary Fig. S1 in the Supplementary Information.

#### Inferential statistical analysis

Quantification of oscillatory measures for inferential statistics was carried out by a cluster-based approach based on randomization (Maris and Oostenveld 2007). This approach identifies clusters (in time, frequency, and space, i.e. electrodes) of activity based on whether the null hypothesis can be rejected while addressing the multiple-comparison problem. The randomization distribution was computed after 1,000 permutations of the independent variable (i.e. attention left vs. right or baseline vs. task) and *t*-test for dependent samples was used as test statistic. At each iteration, the sum of the *t*-values of the largest observed cluster was computed (cluster alpha threshold at 0.05). The original contrast was compared against this randomization distribution at an alpha level of 0.05, Bonferroni corrected for each tail of the distribution. Relationships between behavioral (RT) and neural data (tuning response) were examined using correlations within the cluster-based permutation framework. Rain cloud plots (Allen et al. 2019) were utilized for data visualization when appropriate.

### Experiment 2

#### Participants

Fourty volunteers were recruited at the local university (mean age M ± SD 25.17 ± 7.52 years, 18 female). All participants gave written informed consent in accordance with the Declaration of Helsinki prior to participation. The study was approved by the University of Zürich ethics committee.

#### Stimulus material and procedure

A dichotic listening task, an auditory version of the delayed matching to sample task, was programmed within MATLAB 2016b, using the PsychToolbox. Participants were instructed to maintain central fixation throughout the experiment. After a baseline period (3,000 ms, Fig. 7A), an auditory cue (100 ms duration; 440 Hz) was presented randomly either to the left or to the right ear via headphones. Following an interstimulus interval of 2,000 ± 500 ms, the syllables “goeb” and “goed” were presented binaurally for 500 ms. During a retention interval of 2,500 ms, participants were asked to keep central fixation and maintain the particular syllable presented in the cued ear. Finally, a probe consisting of the binaural presentation of the 2 syllables was presented. Participants were asked to indicate whether or not the 2 consecutive syllables in the cued ear were identical or different. Responses were given via numeric pad with 1 (same, left index finger) and 3 (different, right index finger). The experiment consisted of 100 trials (50 per location left/right ear) with randomized cue and syllable occurrence.

#### Data acquisition

A 128-channel EEG system (Geodesic HydrocCel system, Electrical Geodesics, Eugene, Oregon, USA) was used. Prior to sampling at 500 Hz, the EEG was filtered using a 0.1 Hz high-pass and a 200 Hz low-pass hardware filters. The vertex (Cz) electrode served as a recording reference. Electrode impedances were kept below 40 kΩ. Electrodes around the cheeks and neck were excluded from subsequent analyses. The discarded electrode labels were E1, E8, E14, E17, E21, E25, E32, E48, E49, E56, E63, E68, E73, E81, E88, E94, E99, E107, E113, E119, E125, E126, E127, and E128. After a band pass filtering 1-45 Hz bad electrodes were detected and excluded using the neighbor correlation method implemented in *ft_badchannel* included in the FieldTrip toolbox. The correlation threshold was set to 0.5 after which the data were converted to a common reference. Interim conversion to EEGLAB (Delorme and Makeig 2004) allowed ICA decomposition and exclusion of components associated with ocular, cardiac, and muscle activity by the automatic routines provided by the IClabel plugin (https://labeling.ucsd.edu/tutorial/overview). Subsequently, after converting back to FieldTrip, missing electrodes were interpolated using spline interpolation.

#### Eye tracking

A video-based eye-tracker was used to monitor eye movements (EyeLink 1000 Plus, SR Research, http://www.sr-research.com). Prior to EEG recording, eye tracker calibration consisted of 9 points randomly appearing on the visual display. Participants were instructed to keep their gaze on a given point until it disappeared. A first run served as calibration and a second as validation. If the average error of all points (calibration vs. validation) was below 1° of visual angle, the positions were accepted. Otherwise, calibration was redone until this criterion was reached. The eye-tracker had a sampling rate of 500 Hz and an instrumental spatial resolution of 0.01. The movements of the left eye were tracked.

#### Eye tracking data analysis

The eye-tracking and EEG datasets were synchronized with the EYE-EEG toolbox (Dimigen et al. 2011). For each trial, corresponding time courses of horizontal and vertical eye position were extracted and concatenated resulting in two vectors of 1*×* sample points. A 2D density histogram was created after multiplying each data point (e.g. horizontal and vertical position) with a gaussian filter following the procedures reported here (https://stackoverflow.com/questions/46996206/matlab-creating-a-heatmap-to-visualize-density-of-2d-point-data). The resulting density plot was converted into a MATLAB structure that can be used within FieldTrip. Statistical evaluation of gaze density was carried out within the cluster-based nonparametric framework described above.

Frequency and statistical analyses were similar to Experiment 1.

## Results

During EEG acquisition participants were cued to a particular speaker location. After a delay interval, during which maintenance of the cued location was required, a target was presented at the cued speaker. Participants were asked to indicate via button press whether they heard the syllable “goeb” (left button press) or “goep” (right button press) (Fig. 1A and B). Response times (RT) did not vary with speaker location (Fig. 1C) and the overall hit rate was 96.3% ± 8.3% (M ± STD). Hit rate for left and right loudspeakers respectively was 96.1% ± % ± 8.2% and 96.4% ± % ± 8.7% (*t*_30_ = −0.83, *P* > 0.4). Behavioral results confirm the participant’s task compliance and indicate no behavioral bias towards any particular speaker location.

The auditory cue presentation was associated with reliable event-related potentials (ERPs) with a typical auditory scalp topography characterized by the largest negativity of the N100 ERP components around the vertex electrode (Fig. 2A). Source reconstruction confirmed an origin in the vicinity of the left primary auditory cortex for right cues and the right primary auditory cortex for left cues (Fig. 2A). However, the difference in neural generators in the interval 110–180 ms associated with left versus right spatial cue processing was distributed across bilateral higher order auditory and parietal brain areas (Fig. 2B). Processing of left auditory cues was associated with a stronger neuronal response in the right parietal cortex contralateral to the cued direction and vice versa.

**Fig. 3:**
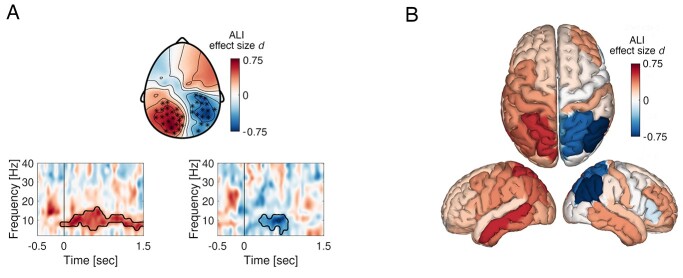
Alpha activity in posterior parietal cortex is modulated during the maintenance of the spatial position of auditory cues. (A) Time–frequency representation of power illustrating the contrast (Alpha Lateralization Index; ALI converted in Cohen’s *d* effect size) between left and right presented stimuli. Time is depicted on the *x*-axis and frequency on the *y*-axis. The variation of ALI expressed in effect size is color-coded. Marked electrodes in the topography correspond to the cluster of electrodes confirming significant condition differences in alpha power (cluster permutation approach, *P* < 0.025). (B) Source reconstruction of the contrast in (A) illustrating the involvement of posterior-parietal brain areas.

The lateralization in neuronal activity was also apparent when analyzing the data in the time–frequency domain (Fig. 3). Maintenance of auditory cues to the left was associated with a contralateral decrease in alpha power (Fig. 3A, *P* < 0.025, cluster permutation test, effect size Cohen’s > ± 0.6) and a relative increase in the ipsilateral hemisphere. Condition differences in alpha power were present in both hemispheres predominantly around the time window 300–800 ms after cue onset. Hence, source analysis was centered around this time window. Source analysis confirmed lateralized activation pattern involving parietal brain areas (Fig. 3B), largely resembling the distributed activity observed in the time-domain source analysis (Fig. 2B). In summary, both time and time-frequency domain analyses confirmed the hypothesis that the modulation of neuronal activity in the posterior parietal cortex reflects the maintenance of auditory spatial information. However, it is thus far unclear whether this alpha modulation is associated with only a coarse left versus right differentiation or whether the engagement by auditory attention exploits the spatial high fidelity of the posterior parietal cortex. For this purpose, we aimed to decode the speaker location based on the alpha oscillatory activity.

**Fig. 4:**
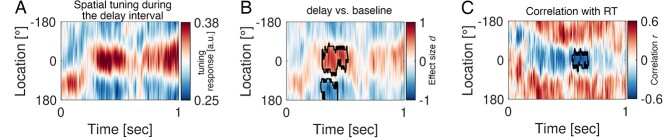
Alpha activity induced by auditory cues reflects the neural encoding of space. (A) The tuning response as a function of time-averaged across participants. The *x*-axis denotes time with cue onset at 0 s and the *y*-axis illustrates spatial location ranging from −180° to 180° (see methods). Illustrated is a contrast of post cue onset tuning response (0 to 1 s) against pre cue tuning response of equal length. Hence, the time axis range from 0 to 1 s post cue onset. Maximum tuning response (reflected by warm colors) at location 0° corresponds to a strong link between alpha activity and the encoding of spatial information. (B) Same as in (A) but expressed in units of effect size Cohen’s d derived from the contrast against the pre cue onset baseline of equal length (cluster permutation test, *P* < 0.025). The black contour line highlights the time × location cluster supporting the rejection of the null hypothesis (neural tuning data during baseline and delay intervals do not differ). (C) Correlation between RT and the tuning response.

A forward encoding modeling approach (see Materials and methods) was utilized to decode the direction of the cue from the multivariate data in the alpha band (Fig. 4). Throughout the delay interval, a robust tuning response to loudspeaker location was observed with a peak latency between ~300 and 800 ms after cue onset (Fig. 4A). This tuning was specific to the delay period as confirmed by a cluster permutation test when compared to a pre cue baseline of equal length (i.e. 1,000 ms, Fig. 4B, cluster permutation test, *P* < 0.025). Estimated channel response profiles as a function of position are provided in Supplementary Fig. S2 (Supplementary Information). Tuning response data were related to reaction time (RT) utilizing correlation as the test statistic during the cluster-based permutation approach (Fig. 4C). Participants with strong tuning to speaker location during the delay interval were faster in responding to the target several seconds later. Note that, these clusters cannot be interpreted in terms of their specificity for a particular latency and/or tuning location. Instead, all of them equally support the rejection of hypothesis H0 (tuning response values during baseline and activity stem from the same distribution). In summary, analyzing power modulations of alpha activity can reliably decode the loudspeaker location towards which individuals attend, beyond the left–right locations.

**Fig. 5:**
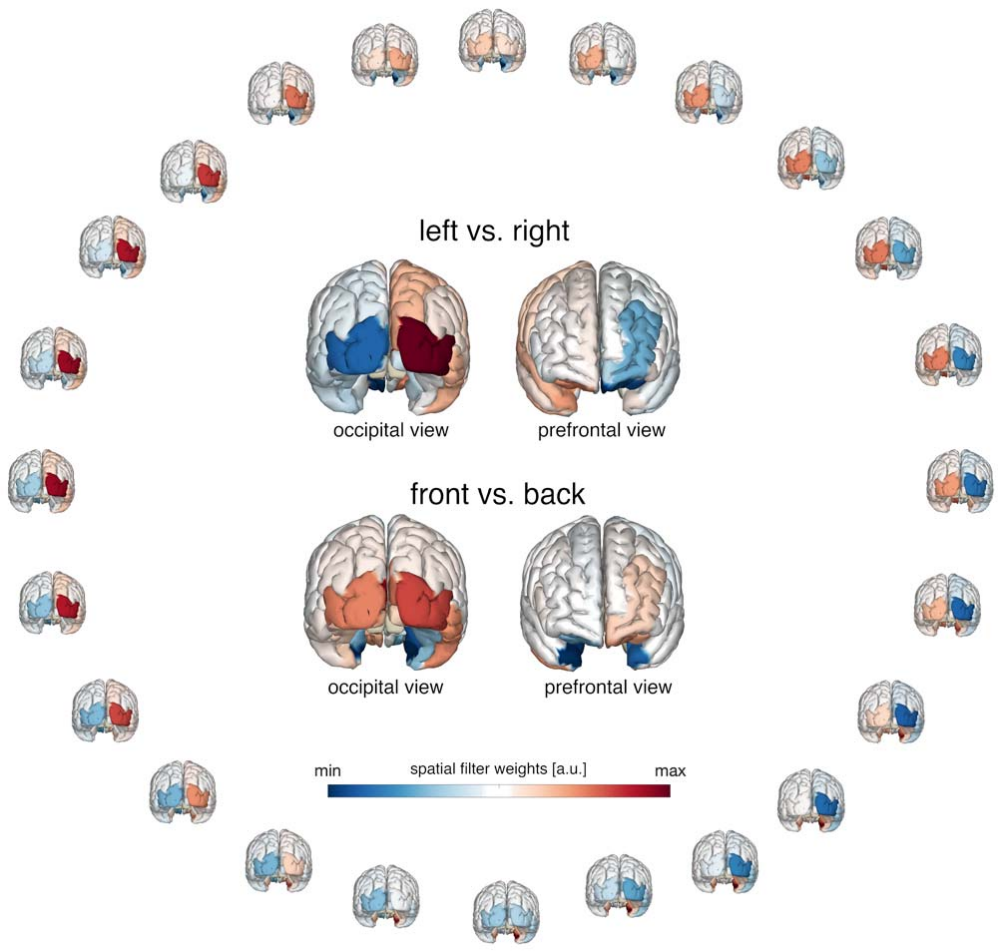
Retinotopic activation of alpha activity in parieto-occipital cortex supports encoding of spatial auditory cues. Distribution of the sources of the alpha power on the cortical surface reflecting the attended direction derived from spatial filter weights obtained from the forward encoding model (activation patterns *A*, see methods). Warm colors indicate a relative alpha band increase and cold colors a decrease expressed in arbitrary units. Insets illustrate the difference between left minus right loudspeaker location (top) and front minus back loudspeaker direction (bottom).

Mapping activation patterns (“*A*”; see method section) onto the cortical surface revealed that the tuning response was mainly driven by activity originating from the visual and parietal cortex (Fig. 5). Despite a clear auditory task demanding encoding, maintenance, and processing without relying on visual material, brain areas previously associated with the processing of visual information display “retinotopic” organization during audition.

**Fig. 6:**
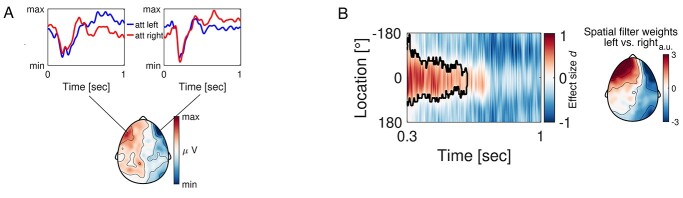
Saccades are consistent with the location maintenance of auditory cues. (A) The topography illustrates the ERP difference during the delay interval (300–1000 ms, avoiding the first 300 ms dominated by the auditory ERP) between left versus right auditory cue location. The time courses reflect the grand average ERP from a representative left (“E48”) and right (“E221”) electrode. The red time course denotes attention directed towards the right loudspeaker locations and the blue time course towards the left. (B) The tuning response as a function of time-averaged across participants utilizing not artifact corrected time domain signals. The *x*-axis denotes time with cue onset at 0 s and *y*-axis illustrates spatial location ranging from −180° to 180°. As illustrated in (A), the first 300 ms are dominated by a strong ERP. For this reason, the *x*-axis range from 0.3 to 1 s was chosen for analysis in order to avoid contamination by this evoked activity. Maximum tuning response (reflected by warm colors) at location 0° corresponds to a strong link between saccade direction and the encoding of spatial information. Tuning response is expressed in units of effect size (Cohen’s *d*) derived from the contrast against the pre cue onset baseline of equal length (cluster-permutation test, *P* < 0.025). The black contour line highlights the time × location cluster supporting the rejection of the null hypothesis (neural tuning data during baseline and delay intervals do not differ). The scalp topography illustrates the distribution of the spatial filter weights obtained from the forward encoding model (activation patterns *A*, see methods), consistent with the saccade topography depicted in (A).

The most informative brain regions are clearly visual. The question arises, why visual cortex activity will contribute to task engagement and processing during the auditory task? As vision is not a required sensory modality, a possible interpretation can be derived only from a multimodal perspective. While a sensory approach would argue for a direct effect of auditory processing on posterior regions (e.g. Cohen et al. 2005), recent literature suggests that action-related sensory input mediates multisensory effects. For example, eye movements during auditory attention inform individual group differences within the dorsal attention network (Braga et al. 2016), and eye-movement-related eardrum oscillations link sounds and images in space (Gruters et al. 2018; Murphy et al. 2020). Thus, alternatively, an affirmative case for the presence of saccades in register of auditory cue location might offer some explanation. We conducted an exploratory analysis re-evaluating the epoched data prior to ICA correction. As an eye-tracking device was not available, we reasoned that if aspects of oculomotor activity are present during the delay interval, these will be reflected in the EEG topography. Specifically, if the saccade direction is consistent towards the direction of the cued position, the difference in ERP topography (left–right) should be characterized by a prototypical saccade topography. The results of this analysis are illustrated in Fig. 6A. The topographic difference in the interval 300–1,000 ms post auditory cue onset between attention directed towards left speaker location (red) versus right speaker location (blue) displays a clear oculomotor topography. The ERP time courses are derived from a representative left frontal electrode (“E48”) and right frontal electrode (“E221”), respectively. The position of these electrodes corresponds to the approximate position of a hEOG. Using this time-domain data containing saccadic activity, we performed the forward encoding procedure described above. Indeed, an increase in tuning response towards different speaker locations as compared to pre cue baseline was apparent (Fig. 6B, cluster-permutation test, effect size Cohen’s *d* > 0.5). That is, the variation of saccades during the maintenance interval of the auditory cue was not random but, in a direction, consistent with the cued speaker's location. Moreover, the topography of the spatial filter weights resamples the oculomotor topography illustrated in Fig. 6A. This suggests that auditory attention is linked to the visual system, at least in part, through pro-active orientation towards the relevant sound origin via saccades towards the sound source.

Motivated by these observations that a) alpha power lateralization during auditory spatial attention recruits visual cortical areas and b) visual cortex activity might be instantiated by location-consistent oculomotor activity, a confirmatory experiment was conducted utilizing both eye tracking and simultaneous EEG. Following the results in Experiment 1, it remains unclear, to what extent they can be interpreted in the context of active attention deployment. It is in principle possible that during the delay interval, participants do not necessarily maintain the spatial position of the cue (as it was always correct) and do not actively direct attention towards the cued location. That is, the implicit assumption that the spatial cue guides the participants’ attention is challenged by the alternative of an automatic orientation response. Instead of an active direction of attention, this automatic response is an equally suitable interpretation of the present findings. Furthermore, in the first auditory experiment, the speakers were visible to the participants, which by itself can provide some important visual cues to saccade to. In turn, this could partly explain the oculomotor activity reported in Fig. 6. To address this, we reasoned that, in a dichotic listening task, vision is not a necessary sensory modality and in principle should therefore not aid in task performance. In contrast, however, if the eye-movement pattern is associated with modulation of alpha activity, lateralized alpha power should result in a lateralized pattern of eye-movements despite their limited benefit for task performance. Hence, such an outcome will provide empirical support for the notion that posterior alpha power lateralization reflects a bottom-up orientation response associated with consistent biases of gaze direction, and it can, but does not necessarily depend on, the active top-down attention deployment.

**Fig. 7:**
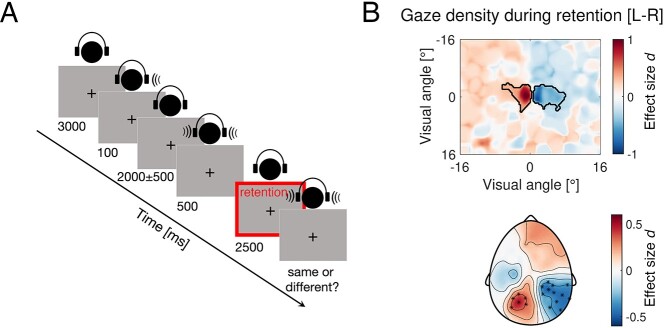
Alpha power lateralization is accompanied by lateralized gaze direction. (A) Schematic illustration of a representative trial in the auditory dichotic listening task. (B) Top: condition difference in gaze density (L vs. R) during the retention period highlighted with a red bounding box in (A). Black outline highlights clusters of significant differences in gaze direction after cluster-permutation test (*P* < 0.025). Bottom: topography of alpha power lateralization during the retention interval expressed in units of effect size (Cohen’s *d*). Highlighted electrodes correspond to clusters identified after the cluster-permutation test (*P* < 0.025).

Participants were asked to maintain central fixation throughout the experiment while sitting in a dimly lit room with their head positioned on a chin rest. Stimuli were delivered via headphones. An auditory cue was presented randomly either in the left or right ear, signaling the relevant site/direction. Participants are required to encode binaurally presented syllables “goeb” or “goed” and retain the one presented in the cued ear. After a retention interval of 2,500 ms, the stimuli were presented again and participants were asked to indicate whether or not the syllable in the cued ear was identical, or different from the previously encoded one (Fig. 7A). Posterior alpha power was lateralized during the retention period of an auditory stimulus (Fig. 7B, cluster permutation test *P* < 0.025, effect size Cohen’s *d* > ±.5). Crucially, analysis of the gaze direction during the same retention interval revealed reliable lateralization as well (Fig. 7B, cluster permutation test *P* < 0.025, Cohen’s *d* > ± 1). Alpha power contralateral to the gaze direction was found to be reduced and vice versa. The strongest effect in gaze direction density was found within the range of ±2° of visual angle. A range that typically falls within the one considered as a fixation and is likely not accounted for during traditional artifact control analyses that exclude scalp topographies associated with oculomotor activity such as saccades and eye blinks. Furthermore, this association between alpha power lateralization and gaze bias is preserved even under conditions where top-down attention is required for task performance (van Ede, Chekroud, Stokes, Nobre 2019) and the presence and absence of microsaccades is controlled (Liu et al. 2022). In this latter report by Liu and colleagues, the authors convincingly demonstrate that microsaccades are not a necessary condition for the modulation of alpha activity during spatial attention tasks. A re-analysis of this open dataset replicated the main finding that alpha power lateralization with spatial attention is a robust phenomenon even in the absence of saccades towards the attended hemifield (Supplementary Fig. S3). And yet, the lateralization of gaze bias during the lateralization of alpha power was preserved (Supplementary Fig. S3). Finally, this association between the lateralization of gaze and alpha power is still preserved (Supplementary Fig. S4) even in cases where eye tracker information is utilized to monitor the participants’ gaze and lateralized stimuli are only presented during fixation maintenance (Schindler et al. 2022). That is, Schindler and colleagues have controlled for this by “pausing the presentation of faces whenever the gaze was not directed at the center of the screen (0.6° around the center)” (p. 5 Schindler et al. 2022).

Taken together, the present results and the re-analyses of openly available data support the conjecture that lateralization of posterior alpha power entails, at least to some extent, a bottom-up orientation response reflected in consistent gaze shifts towards the spatial location of the cued attention. We conclude that spatial attention, both auditory and visual, leads to oculomotor action in direction of the attended location and concomitant lateralization of posterior alpha power.

## Discussion

Navigation in a complex environment requires the integration of multiple sensory streams. Research has discovered a variety of supramodal brain areas responding to input from different sensory modalities. The present report provides empirical support for a supramodal neural circuit in service of spatial attention reflected by the spatial distribution of alpha-band activity. The activation patterns resembled those known from visual–spatial attention studies and demonstrate a supramodal topographic organization with respect to the direction of attention, initiated at least in part through oculomotor action. Based on these patterns of neuronal activity in the alpha band, we demonstrate that the maintained spatial direction of the cue can be decoded, where stronger spatial tuning was associated with faster responses.

### Impulses for the multimodal view of the brain and the role of alpha power lateralization

Present results open novel empirical questions both in the fields of visual and auditory attention but also directed towards our current understanding of the multimodal brain.

The involvement of eye-movements towards the attended loudspeaker location (Experiment 1) and cued ear (Experiment 2) provides evidence for the existence of a reciprocal relationship as the recently discovered saccades induced eardrum oscillations (Gruters et al. 2018; Murphy et al. 2020) and consistent modulation of neural excitability within auditory areas by saccadic eye movements (Leszczynski et al. 2022). That is, an auditory cue presentation at a particular location in space elicits oculomotor responses consistent with the sound origin.

Noteworthy, artifact control of eye movements eliminates the muscular contribution to the EEG scalp topography. Yet the consequence of the movement registered by the sensory system, i.e. alpha power modulation contralateral to gaze direction, remains unaltered (e.g. Experiment 2). Two spatially distinct topographic patterns associated with the cortical representation of auditory space have been discussed previously: a frontal lateralization in the delta frequency range (0.02–2 Hz) and a posterior alpha lateralization (Bednar and Lalor 2018). While it is tempting to interpret these patterns as “cortical activity tracks the time varying azimuth of moving sound” (p. 689 in (Bednar and Lalor 2018)), present observations suggest an alternative. The frontal lateralization pattern in the delta range (e.g. Fig. 3 in (Bednar and Lalor 2018)) is reminiscent of the oculomotor activation pattern in Fig. 6A and B. It is conceivable therefore that variation of gaze direction with sound location gives rise to both: frontal topography capturing eye-movement activity in the low-frequency range and posterior alpha lateralization reflecting the registration of the movement by the visual system.

The present results by no means challenge or invalidate previous work on auditory spatial attention. Quite contrary, motivated by this work we provide complementary value towards the interpretation of earlier findings suggesting the incorporation of eye movements as a signal (e.g. behavioral outcome) rather than an artifact. In line with recent conclusions that alpha oscillations do not alter excitability in the visual cortex (Zhigalov and Jensen 2020) and do not seem to suppress irrelevant external input during spatial selection (Foster and Awh 2019), the present association between the consistency in eye movements and alpha oscillations offers a new direction for experimental and theoretical development of existing models on the role of alpha oscillations in cognition (Klimesch et al. 2007; Jensen and Mazaheri 2010). In particular, shifts in gaze direction are an integral part of psychological constructs such as “internal selective attention” as recently highlighted in (van Ede and Nobre 2022), and potentially offer novel testable predictions towards the biological manifestation of psychological phenomena.

### Alpha power modulation allows decoding of auditory covert attention

In visual spatial attention, a large body of evidence suggests that the direction of attention can be decoded based on posterior alpha activity (Foster et al. 2016; Samaha et al. 2016; Foster et al. 2017; Popov et al. 2017; Munneke et al. 2019) using forward encoding models (Brouwer and Heeger 2009). Here we confirm that this finding generalizes to the auditory domain, as noted previously (e.g. Bednar and Lalor 2018), and extend to directions beyond the visual field (i.e. to the sides and behind the participant). That is, posterior alpha power modulation does not simply reflect suppression of anticipated interfering visual input. Instead, it reflects an active process of tuning to sound origin and directing attention to optimize performance (e.g. faster RTs correlated with stronger tuning; Fig. 4). To what extent this tuning is specific to alpha oscillations, eye-movement control, or their interaction merits further examination. In Experiment 2, we have demonstrated that the lateralization in gaze direction scales with the lateralization of alpha power. This is in line with observations that recall of an item stored in visual working memory is associated with a consistent gaze pattern in the direction of the memorized location (van Ede, Chekroud and Nobre 2019), a gaze pattern that differentiates future item selection (van Ede et al. 2021) and is conceived as an oculomotor signature of attention in service of memory-guided behavior (van Ede et al. 2020). Future work should refine the relationship between gaze direction, alpha oscillations, and the tracking of spatial representations in working memory, as gaze shifts are present even in cases where microsaccade influence is eliminated (see Supplementary Fig. S3 and Liu et al. 2022).

## General conclusion

In conclusion, the present results confirm a multimodal functional relevance of alpha oscillatory activity that reflects the integration of auditory and visual utilities of the observing individual into a direction-specific sensorimotor gain increase to organize and instantiate coordinated behavior.

## Supplementary Material

Supplementary_information_bhac285
