# Supplementary material for: Brain areas associated with visual spatial attention display topographic organization during auditory spatial attention
Source: Cereb Cortex. 2022 Aug 16;33(7):3478–89. doi: 10.1093/cercor/bhac285 (PMC10068281; doi:10.1093/cercor/bhac285)
Supplement: Supplementary_information_bhac285 [file supplementary_information_bhac285.docx]

**Supplementary information**


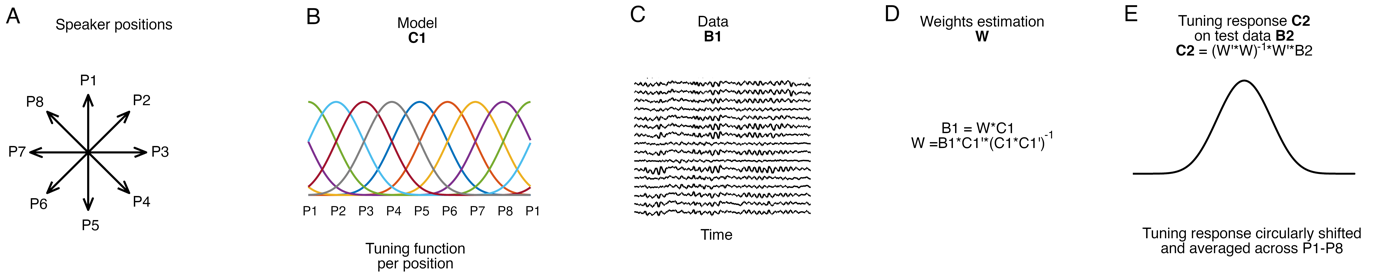


**Figure S 1:A**- For simplicity, the positions of the speakers are reduced to eight. Each speaker location is assigned a position (P1 to P8). **B-** A model (C1) of hypothetical tuning functions that span the entire position space. Each tuning function has a high sensitivity for a particular position**. C-** EEG recording is used as a training data set B1. **D**- Spatial tuning weights are derived utilizing the tuning model and the B1 training data set. **E-** Hypothetical tuning response denoted as C2 derived from test dataset B2 and the spatial weights W. Under ideal conditions, the shape of the tuning functions in the model (B) and the computed tuning response (E) are identical. Thus, averaging across all tuning responses after a circular shift toward a common center results in a bell-shaped curve.


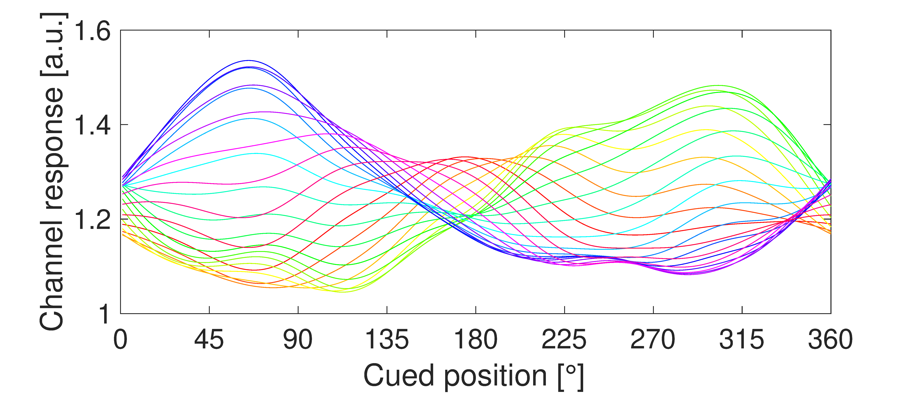


**Figure S 2:** Estimated channel response profiles as a function of cued speaker location.


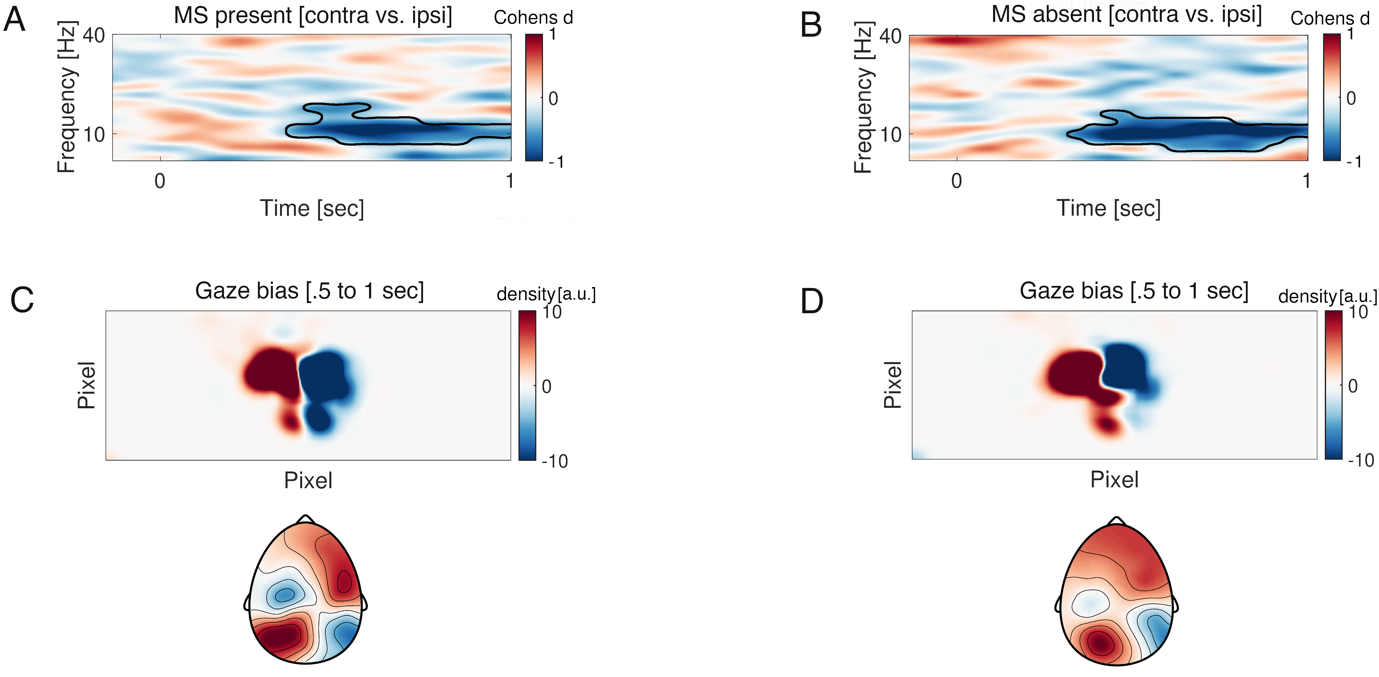


**Figure S 3: A-B**: Time-frequency representation of power (contra vs. ipsi) over occipital electrodes. Black outline highlights the clusters of activity supporting the rejection of H0 after cluster permutation test. Illustration is identical to the Figures 2A and B in Liu et al. 2021. **C:** Gaze bias density during the condition where micro saccades towards the cued location were present (MS present) for the contrast left minus right attention direction during the time interval of 500-1000ms with strong alpha power lateralization (scalp topography of 8-12 Hz activity, bottom) **D:** same as C but for trials with absent micro saccades towards the cued location.


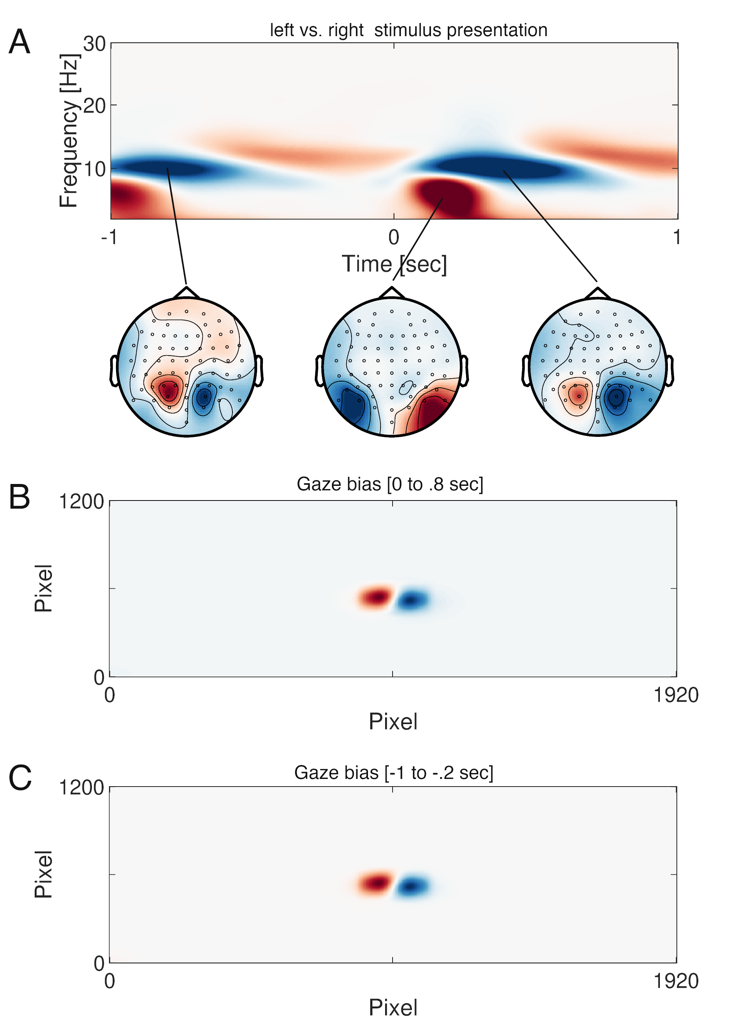


**Figure S 4: Reanalysis of Schindler et al. 2022 A**- Replication of the main result presented in Figure 6 of the original publication: alpha power is lateralized before and after lateralized stimulus presentation. Time-frequency representation of power (contra vs. ipsi to face persentation ) over occipital electrodes. **B:** Gaze bias density during the time interval of 0-800ms with strong alpha power lateralization (scalp topography of 8-12 Hz activity, right topography in A) **C:** same as B but for the interval -1000 to -200ms before stimulus presentation.
